# Tick-Borne Hemoparasites of Sheep: A Molecular Research in Turkey

**DOI:** 10.3390/pathogens10020162

**Published:** 2021-02-03

**Authors:** Onur Ceylan, Benedicto Byamukama, Ceylan Ceylan, Eloiza May Galon, Mingming Liu, Tatsunori Masatani, Xuenan Xuan, Ferda Sevinc

**Affiliations:** 1 Department of Parasiyology, Faculty of Veterinary Medicine, Selcuk University, 42250 Konya, Turkey; onurceylan@selcuk.edu.tr (O.C.); ceylanilhan@selcuk.edu.tr (C.C.); 2 National Research Center for Protozoan Diseases, Obihiro University of Agriculture and Veterinary Medicine, Obihiro, Hokkaido 080-8555, Japan; benards.benedicto4@gmail.com (B.B.); eloizagalon@gmail.com (E.M.G.); lmm_2010@hotmail.com (M.L.); 3 Faculty of Applied Biological Sciences, Gifu University, Gifu 501-1193, Japan; tatsunorimasatani@gmail.com

**Keywords:** *Babesia ovis*, *Theileria ovis*, *Anaplasma ovis*, *Anaplasma phagocytophilum*, sheep

## Abstract

Tick-borne diseases (TBDs) indulge in severe economic losses in the livestock industry by adversely affecting the small ruminant breeding in tropical and subtropical zone countries, including Turkey. Turkey encompasses a wide land area representing diverse climatic conditions. The present study explored the presence and distribution of *Babesia ovis*, *Theileria ovis*, *Theileria lestoquardi*, *Anaplasma ovis*, *Anaplasma phagocytophilum* and the co-occurrence status of these pathogens. A total of 299 sheep blood samples were collected from fifteen provinces located in six different geographical regions in Turkey. PCR analyses were executed using species-specific primers based on *Babesia ovis* BoSSU rRNA, *Theileria ovis* ToSSU rRNA, *Theileria lestoquardi* 18S rRNA, *Anaplasma ovis* Major Surface Protein *(AoMSP4)*, and *Anaplasma phagocytophilum* 16S rRNA genes. Overall, 219 (73.24%) sheep were found to be infected with at least one of the following protozoan and rickettsial pathogens; *B. ovis*, *A. ovis,* *T. ovis*, and *A. phagocytophilum*. *Theileria lestoquardi* was not detected in any blood sample. The global prevalence of *B. ovis*, *A. ovis, T. ovis*, and *A. phagocytophilum* was estimated to be 2.68%, 16.05%, 41.47%, and 57.19%, respectively. Besides this, dual (24.41%), triple (9.03%), and quadruple (0.67%) co-infections were detected in the study. Phylogenetic analysis revealed significant nucleotide sequence identities between the sequences obtained in this study and the sequences registered in the GenBank. This study provides relevant data regarding the predominance of ovine tick-borne protozoan and rickettsial agents in Turkey. A high molecular prevalence of tick-borne pathogens (TBPs) was identified in the study. This situation indicates that TBPs should be screened continuously, and necessary control measures should be taken to prevent diseases caused by tick-borne protozoan and rickettsial agents.

## 1. Introduction

In the veterinary field, tick-borne diseases (TBDs) are highly significant [1]. Tick-borne hemoprotozoan and rickettsial agents hinder animal production in tropical and subtropical zone countries, including Turkey [2]. These agents negatively affect small ruminant breeding by reducing the yield and causing abortions. This, in turn, leads to serious economic losses in the livestock industry [2,3,4].

Several hemoparasitic and rickettsial diseases transmitted by vector ticks could infect small ruminants in Europe [5]. Among these, anaplasmosis, babesiosis, and theileriosis are the main TBDs and pose threats to the small ruminant breeding and livestock economy [1]. As in some other countries [6,7], the small ruminant breeding is also one of the primary sources of milk and meat in Turkey, which has a geographically advantageous position between Asian and European countries comprising approximately forty million sheep and favorable climatic conditions for vector ticks [2,8].

The most economically significant hemoprotozoan parasites of small ruminants, *Babesia* spp. and *Theileria* spp., have worldwide distribution [1]. While ovine babesiosis is responsible for an acute disease characterized by fever, hemolytic anemia, hemoglobinuria, and icterus, ovine theileriosis is characterized by lymphoproliferative disease with high mortality and morbidity. Malignant ovine theileriosis incurs deaths in the Mediterranean region, Middle East, South East Asia, and the Indian subcontinent [9,10]. It was detected in Europe [11] and the neighboring countries of Turkey (Iran, Iraq) [12,13]; however, it has not been reported in sheep from Turkey [14].

The species of the genus *Anaplasma* (Rickettsiales: *Anaplasmataceae*) are tick-transmitted obligate intracellular microorganisms affecting both human and animal health [10]. *Anaplasma centrale*, *A. marginale*, *A. bovis*, *A. ovis*, *A. platys*, and *A. phagocytophilum* are the most known etiological agents of anaplasmosis [15]. Several *Anaplasma* species, including *A. ovis, A. marginale*, and the human granulocytic anaplasmosis (HGA) agent *A. phagocytophilum*, can cause ovine anaplasmosis [10]. It was also reported that *A. ovis* might be a zoonotic rickettsial pathogen [16,17]. Although *A. ovis* generally cause subclinical infections in sheep, severe diseases characterized by hemolytic anemia can occur in stressful conditions [18]. *Anaplasma phagocytophilum*, formerly known as *Ehrlichia phagocytophila* and *E. equi*, is particularly transmitted by *Ixodes ricinus* and causes tick-borne fever (TBF) with symptoms including fever, anorexia, dullness, and sudden drop in milk yield in domestic ruminants. Reduced weight gain, abortions, and fertility disorders have also been documented [3,4,19].

Several molecular studies regarding ovine TBDs were conducted in Turkey [20,21,22,23]. However, most of these studies geographically restricted to only one region or province. Aouadi et al. [6] and Ringo et al. [24] stated that studies including larger sample size and wider geographical areas could provide deeper insights regarding the actual prevalence and economic importance of tick-borne pathogens (TBPs). Therefore, the aim of this study was the molecular survey of tick-borne hemoprotozoan and rickettsial agents leading to various economically significant yield losses such as meat, milk, manure, skin, and wool in Turkish small ruminants grazing in fifteen different provinces, with some agents being zoonotic as well.

## 2. Results

### 2.1. Overall Infection Rates

The present study screened 299 sheep for ovine tick-borne hemoprotozoan and rickettsial agents by molecular techniques. Overall, 219 (73.24%) of the 299 sheep were found to be positive. The overall prevalences of *B. ovis*, *T. ovis*, *A. ovis*, and *A. phagocytophilum* were identified as 2.68%, 41.47%, 16.05%, and 57.19%, respectively. *T. lestoquardi* DNA was not found in the samples of the present study. Detailed molecular prevalences according to the provinces are outlined in Table 1.

### 2.2. Co-Infections Detected in the Study

The co-infection rate was estimated to be 46.58% (n: 102) of the total infection number (n: 219). Three types of coinfections (dual (24.41%, n: 73), triple (9.03%, n: 27), and quadruple (0.67%, n: 2)) were reported in the study. Dual co-infections (71.57%) were found to be the most prevalent among the co-infection cases, followed by triple co-infections (26.47%) and quadruple co-infections (1.96%), respectively. Dual, triple, and quadruple co-infections were observed to occur in nine different ways (*B. ovis* + *T. ovis*, *B. ovis* + *A. phagocytophilum*, *T. ovis* + *A. ovis*, *T. ovis* + *A. phagocytophilum*, *A. ovis* + *A. phagocytophilum*, *B. ovis* + *T. ovis* + *A. ovis*, *B. ovis* + *T. ovis* + *A. phagocytophilum*, *T. ovis* + *A. ovis* + *A. phagocytophilum*, *B. ovis* + *T. ovis* + *A. ovis* + *A. phagocytophilum*). Detailed information about co-infections detected in the study is provided in Table 2.

### 2.3. Phylogenetic Analysis

**Figure 1:**
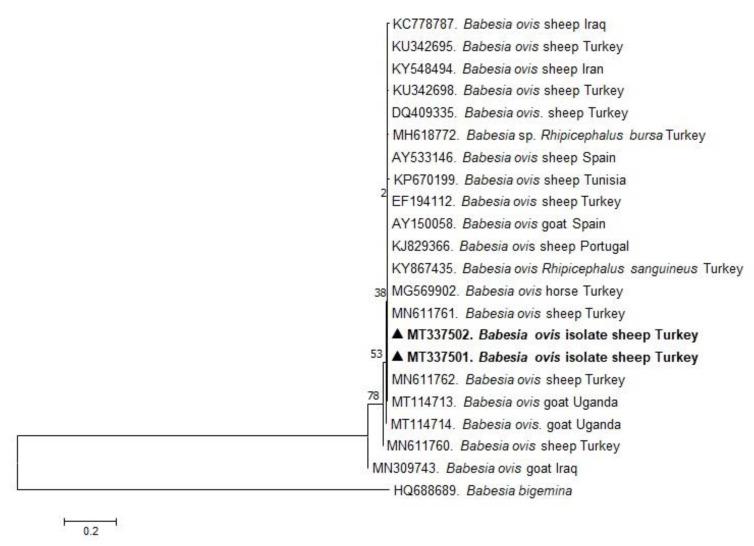
Phylogenetic analysis of the Maximum Likelihood method based on *Babesia ovis* BoSSU rRNA gene sequences. Numbers at nodes represent the percentage occurrence of clades in 1000 bootstrap replications of the data. Sequences from this study are shown in bold font. The 18S rRNA gene sequences of *Babesia bigemina* (HQ688689) are used as outgroup.

**Figure 2:**
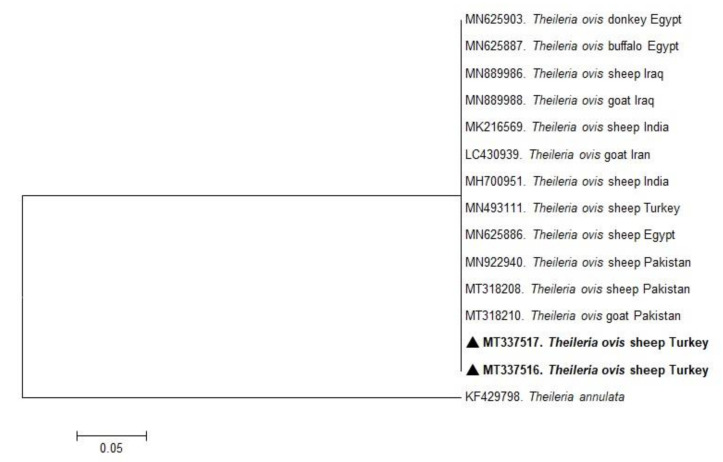
Phylogenetic analysis of the Maximum Likelihood method based on *Theileria ovis* ToSSU rRNA gene sequences. Numbers at nodes reflect the percentage occurrence of clades in 1000 bootstrap replications of the data. Sequences from this study are highlighted in bold font. The 18S rRNA gene sequences of *Theileria annulata* (KF429798) served as outgroup.

**Figure 3:**
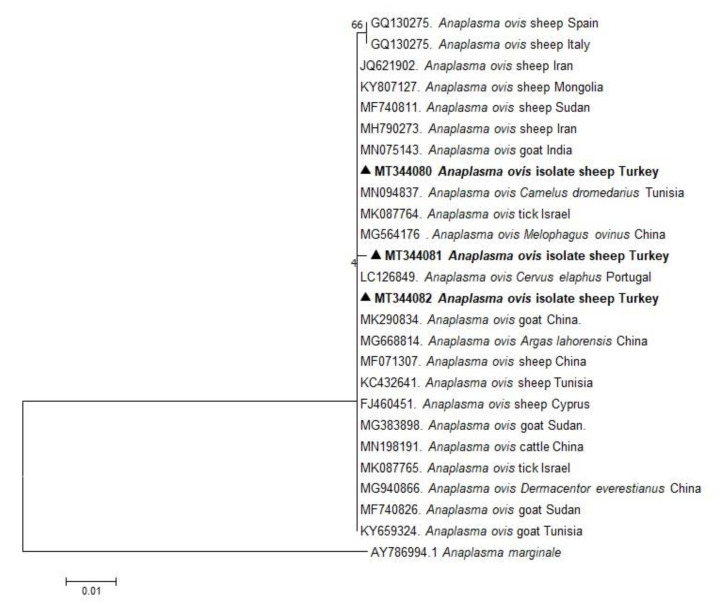
Phylogenetic analysis of the Maximum Likelihood method based on *Anaplasma ovis* MSP4 gene sequences. Numbers at nodes represent the percentage occurrence of clades in 1000 bootstrap replications of the data. Sequences from this study are given in bold font. The MSP4 gene sequences of *Anaplasma marginale* (AY786994) are used as outgroup.

**Figure 4:**
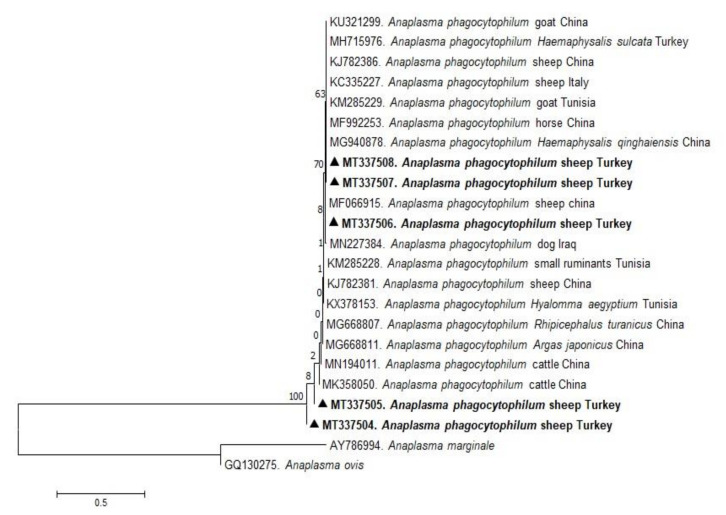
Phylogenetic analysis of the Maximum Likelihood method based on *Anaplasma phagocytophilum* 16S rRNA gene sequences. Numbers at nodes signify the percentage occurrence of clades in 1000 bootstrap replications of the data. Sequences from this study are written in bold font. The MSP4 gene sequences of *Anaplasma phagocytophilum* (AY786994) are used as outgroup.

In the present study, phylogenetic trees of *B. ovis*, *T. ovis*, *A. ovis*, and *A. phagocytophilum* were constructed based on BoSSUrRNA, ToSSUrRNA, MSP4, and 16S rRNA genes, respectively. The sequences of BoSSUrRNA obtained in the study (MT337501, MT337502) established a well-supported clade with the sequences from Turkey and other countries (Figure 1). These sequences revealed 99.10%–100% nucleotide sequence identity with the sequences previously reported from Iraq, Iran, Portugal, Spain, Tunisia, Uganda, and Turkey (99.77%–100%). Accordingly, ToSSUrRNA sequences (MT337516 and MT337516) fell into the same cluster with previous sequences from Turkey and other countries shown on the phylogenetic tree with nucleotide sequence identity value 100% (Figure 2). AoMSP4 sequences (MT344080, MT344081, and MT344082) also formed a well-supported clade with the sequences reported from some African, Asian, and European countries (Figure 3). The nucleotide sequence identity values of AoMSP4 sequences ranged from 99.71% to 100%. *Anaplasma phagocytophilum* 16S rRNA sequences obtained in this study (MT337504, MT337505, MT337506, MT337507, MT337508) clustered together with previously reported sequences from China, Iraq, Italy, Tunisia, and Turkey with the nucleotide sequence identity values 98.17%–100% (Figure 4). The submitted two sequences of *B. ovis* (MT337501, MT337502) from this study showed 100% identity and similarity to each other. This situation was determined as 100% for *T. ovis* sequences (MT337516, MT337516), 99%–100% for *A. ovis* sequences (MT344080-82), and 98%–100% for *A. phagocytophilum* sequences (MT337504-08).

### 2.4. Statistical Analysis

Considering the molecular prevalences of each pathogen in fifteen different provinces, statistical analysis was conducted to elucidate the statistical significance of the differences in molecular prevalence values. The present study established a statistically significant difference when the molecular prevalence values of *A. ovis* (*p* = 0.002, χ^2^ = 33.474), *A. phagocytophilum* (*p* = 0.001, χ^2^ = 49.105), and *T. ovis* (*p* = 0.001, χ^2^ = 73.199) in fifteen different provinces were examined. No statistically significant difference was observed for *B. ovis* (*p* = 0.680, χ^2^ = 11.082) and *T. lestoquardi*. The molecular prevalence of *T. lestoquardi* was the same in all provinces, and no positivity was determined in any city. Further information about statistical analysis is illustrated in Table 3.

## 3. Discussion

Ticks play an important role in veterinary and human medicine owing to their ability to transmit some infectious pathogens, including bacteria, helminths, protozoa, and viruses. It is considered that ticks are the major disease-causing pathogens in wild and domestic animals [25]. In the present study, we screened ovine tick-borne hemoprotozoan and rickettsial agents in sheep from fifteen different provinces located in six geographical regions of Turkey, and *B. ovis*, *T. ovis*, *A. ovis*, and *A. phagocytophilum* were identified in blood samples. However, *T. lestoquardi* was not detected in any of the samples.

Although bovine piroplasmosis attracted great attention in the past years, interest in ovine piroplasmosis attributed to its socio-economic impacts has recently increased [26]. Babesiosis is one of the most significant infections caused by piroplasms and several pathogenic and non-pathogenic *Babesia* species (*B. crassa*, *B. foliata*, *B. motasi*, *B. ovis*, *B. taylori*, *Babesia* sp. (China), and *Babesia* sp. (Xinjiang)) are responsible for ovine babesiosis in small ruminants [27,28,29]. *Babesia ovis* is the most pathogenic species affecting sheep among these species [9] and ovine babesiosis caused by this hemoprotozoan parasite has a country-wide endemic instability in Turkey [30]. It was reported that the seroprevalence of *B. ovis* varied from 35.0% to 74.4% [31]. Although Sevinc et al. [23] estimated an infection rate of 70.81% by molecular techniques in sheep with clinical signs, other molecular studies revealed the infection rate ranging from 0% to 21.42% in randomly selected apparently healthy sheep from different regions and provinces of Turkey [14,22,31,32,33,34]. With some considerable results, the data obtained in this study were in agreement with the outcomes documented in previous studies. Although several serological methods such as Indirect Fluorescent Antibody (IFA) test and recombinant *B. ovis* secreted antigen 1 based Enzyme-Linked Immunosorbent Assay (rBoSA1-ELISA) detected anti-*B. ovis* antibodies in all these provinces [30], molecular detection of *B. ovis* was only reported from Niğde province [14,34]. Present study elucidated *B. ovis* by molecular techniques in sheep for the first time in Ankara, Bartın, Batman, Çorum, Isparta, and Kırşehir provinces of Turkey. As a result of the literature search, no published paper was found. The obtained data can, thus, aid in implementing the envisaged control programs against ovine babesiosis in these provinces in the future.

*Theileria* parasites, transstadially transmitted by ixodid ticks, are the main pathogens of theileriosis in domestic and wild animals [35]. Small ruminants are adversely affected by *Theileria* species resulting in economic losses owing to the infections caused by these hemoprotozoan parasites [36]. Among the *Theileria* species infecting small ruminants, *T. lestoquardi*, *T. luwenshuni*, and *T. uilenbergi* are referred to as the agents of malignant theileriosis, whereas three other species, *T. ovis*, *T. recondita*, and *T. seperata*, are known as non-pathogenic or benign *Theileria* species [35]. Among *Theileria* species, *T. ovis* [37], *T. luwenshuni*, *T. uilenbergi* [14], and some *Theileria* isolates such as *Theileria* sp. MK [21], *Theileria* sp. OT1, and *Theileria* sp. OT3 [14] were detected in Turkey. PCR-based molecular studies reveal that the infection rate of *T. ovis* ranges from 17.0% to 61.4% in different parts of Turkey [31]. However, *T. lestoquardi*, the causative agent of malignant theileriosis, has not yet been obtained in Turkey [14]. In the present study, these pathogenic species were also not detected in any of the blood samples, consistent with the previous studies. The molecular prevalence of *T. ovis* (41.47%) was found to be in accordance with the prevalences determined in the previous studies. Except for Samsun province, located in the Black Sea Region, *T. ovis* was detected in all the other provinces and has been determined to be widely distributed throughout Turkey. Normally, *T. ovis* causes mild infections and, therefore, has less economic significance. However, the hemoprotozoan parasites commonly detected in the present study should not be ignored because severe disease cases can occur under stressful conditions [38].

Several *Anaplasma* species, including *A. ovis, A. marginale*, and the human granulocytic anaplasmosis (HGA) agent *A. phagocytophilum* cause ovine anaplasmosis [10]. *Anaplasma ovis* normally results in mild clinical signs, and *A. phagocytophilum* unfavorably affect the livestock economy leading to various clinical signs with a sudden drop in milk production, abortions, fertility problems, and reduced weight gain especially depending on the stress factors such as animal movement, excessive tick infestation, heat, deworming, co-infection, vaccination, and long-distance transportation [3,4,19,36,39]. Despite these crucial impacts, ovine anaplasmosis is still neglected among TBDs [40]. Molecular investigations of ovine anaplasmosis caused by *A. ovis* have been maintained for decades; however, there are a limited number of studies regarding *A. ovis* infections in Turkey [14,22,23,41,42,43]. Comparing the molecular investigations in Turkey, the prevalence of *A. ovis* infection (16.05%) obtained in this study was found parallel to the prevalence values determined in Southern Turkey (15.9–21.8%) [42], Central Anatolia (14.9%), and South-East Anatolia regions (10.6%) [39]. However, it was found lower than the prevalences determined in East Anatolia (67.35%) [41], Thrace (58.8%) [44], and Central Anatolia regions (56.94%, 64.7%) [22,23] and a study covering a large part of Turkey (63.3%) [14]. Except for Elazığ province, *A. ovis* was detected in fourteen provinces in six of the seven geographical regions of Turkey in this study. The findings concluded by this and other studies validate that ovine *A. ovis* infection is endemic in Turkey. Being one of the human infectious agents, more attention should be given to *A. ovis* because of the widespread distribution in Turkey [16,17]. Renneker et al. [39] reported that *A. ovis* should no longer be ignored, and further studies are essential due to its zoonotic potential and economic impacts on small ruminant breeding.

The first investigation of *A. phagocytophilum* in sheep was conducted in Turkey in 2005, and the presence of antibodies formed against this rickettsial microorganism was detected [45]. It has been determined that the molecular prevalence of *A. phagocytophilum* in randomly selected sheep varied between 0% and 18.90% for fifteen years [14,22,23,41,44,46]; however, Benedicto et al. [43] detected *A. phagocytophilum* DNA in 62.5% of the tick-infested sheep with clinical symptoms in a currently conducted study in Turkey. The prevalence of *A. phagocytophilum* estimated in this study is higher than the previously reported ones but lower than the current report of Benedicto et al. [43]. Considering that the study was carried out in randomly selected sheep, the infection rate of *A. phagocytophilum* is the highest rate detected in sheep in Turkey, and *A. phagocytophilum* was detected in all provinces except for one province (Samsun). This situation indicates that zoonotic *A. phagocytophilum* has a large-scale circulation among sheep and vector ticks and poses a significant infection risk for human beings. *Ixodes ricinus*, the primary vector of *A. phagocytophilum* in Europe, *Haemaphysalis sulcata*, and *Rhipicephalus bursa* were reported to carry this pathogen in Turkey [47,48,49]. Vector competency of the other tick species should be investigated, accounting for the high infection rate of *A. phagocytophilum* in sheep in Turkey.

The co-infection phenomenon of sheep attributed to the ovine piroplasms and *Anaplasma* species has been rarely investigated in Turkey. The causative agents of co-infections of small ruminants have been detected by using molecular techniques in different countries such as Iraq [13], Italy [50], Tunisia [51], Turkey [14,22,23], and South Africa [24]. Overall, 219 (73.24%) of the surveyed sheep blood samples were tested positive for at least one pathogen in this study. The co-infection rate was determined as 46.58% (n: 102) among these infections. Dual, triple, and quadruple co-infection rates were detected as 71.57%, 26.47%, and 1.96%, respectively. These different types of co-infections may adversely affect the prognosis of the TBDs, especially in stressful conditions in comparison to the animals infected with single species [22,23]. Sevinc et al. [23] highlighted a negative interaction between the coexistence of *B. ovis* and *T. ovis* in sheep. Further studies are necessary to explore new interactions, to get information regarding clinical effects, and to specify the best option of therapeutic applications in sheep co-infected with piroplasms and *Anaplasma* species.

In conclusion, we demonstrated the presence of *B. ovis*, *T. ovis*, *A. ovis*, and *A. phagocytophilum* in sheep in fifteen provinces belonging to six of the seven geographical regions of Turkey. Moreover, this study includes some information regarding rarely studied co-infections resulting from ovine tick-borne agents. Dual, triple, and quadruple co-infections were commonly identified in the study. *Anaplasma ovis* and *A. phagocytophilum,* which are of zoonotic importance, were detected as widespread throughout Turkey. The current study will contribute to the data regarding the molecular epidemiology of TBDs in Turkey and the envisaged control strategies of these diseases in the near future.

## 4. Materials and Methods

### 4.1. Study Areas and Sample Collection

**Figure 5:**
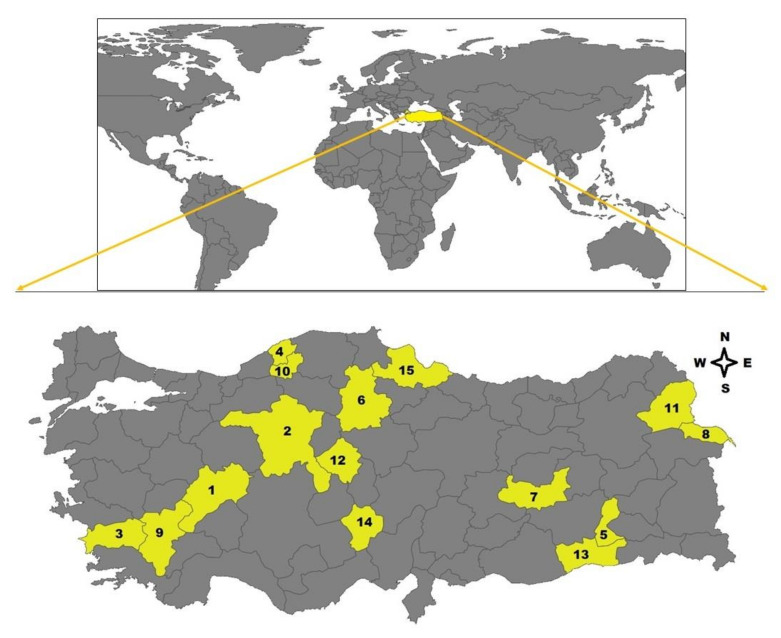
Map of Turkey highlighting fifteen provinces located in the six different geographical regions where the blood samples were collected (1. Afyon, 2. Ankara, 3. Aydın, 4. Bartın, 5. Batman, 6. Çorum, 7. Elazığ, 8. Iğdır, 9. Isparta, 10. Karabük, 11. Kars, 12. Kırşehir, 13. Mardin, 14. Niğde, 15. Samsun).

Whole blood samples (n: 299) of sheep were collected from 15 provinces, including Afyon, Ankara, Aydın, Bartın, Batman, Çorum, Elazığ, Iğdır, Isparta, Karabük, Kars, Kırşehir, Mardin, Niğde, and Samsun located in Aegean (Afyon, Aydın), Black Sea (Bartın, Karabük, Samsun), Central Anatolia (Ankara, Çorum, Kırşehir, Niğde), Eastern Anatolia (Elazığ, Iğdır, Kars), Mediterranean (Isparta), and Southeastern Anatolia (Batman, Mardin) regions of Turkey, between May and August of 2019 (Figure 5). Twenty blood samples were obtained from each city except for Batman (n: 19). The samples were collected from randomly selected apparently healthy sheep raised in small-type farms during the tick activity season. The owners of the sheep were informed about the study and their approval for sampling was received. They stated that the sampled sheep did not receive any ectocid treatment. All sheep were inspected in terms of tick infestation, but no tick infestation was detected in animals.

### 4.2. DNA Extraction

The sheep were individually bled through the jugular vein, and 8 mL blood sample was collected into a tube containing ethylenediamine tetraacetic acid (EDTA) for each animal. Genomic DNA was extracted from the blood samples by using QIAamp^®^ DNA Blood Mini Kit (QIAGEN, Hilden, Germany), following the manufacturer’s instructions. The extracted genomic DNA was stored at –20 °C until the molecular analysis.

### 4.3. Molecular Detection of Tick-Borne Pathogens

*Babesia ovis, T. ovis, T. lestoquardi, A. ovis*, and *A. phagocytophilum* was investigated by species-specific PCR in each DNA sample. The screening was based on the procedures previously described by researchers, respectively listed in Table 4 with their thermocycling conditions. The target genes of species-specific PCR assays were *B. ovis* BoSSU rRNA, *T. ovis* ToSSU rRNA, *T. lestoquardi* 18S rRNA, *A. ovis* MSP4, and *A. phagocytophilum* 16S rRNA as detailed in Table 4. The reaction mixture had a final volume of 10 µL containing 1 µL of 10X ThermoPol Taq Reaction buffer, 0.2 µL of dNTP mix, 0.2 µM of each primer, 0.05 µL Taq DNA polymerase (New England BioLabs, USA), 1.5 µL of DNA template, and 6.85 µL of double-distilled water. Positive controls were set as previously sequence-confirmed DNA templates, and double-distilled water served as negative controls.

### 4.4. Cloning

Following PCR assays, *B. ovis, T. ovis, A. ovis*, and *A. phagocytophilum* amplicons were selected for sequencing. Agarose gel elution was employed to purify amplified PCR products using QIAquick Gel Extraction Kit (Qiagen, Germany), and eluted DNA concentrations were measured on a NanoDrop 2000 spectrophotometer. The extracts were then cloned into a pGEM vector according to the commercial protocol of pGEM^®^-T Easy Vector System (Promega, USA). The template (6 µL) was ligated into pGEM-T easy vector (2 µL) using T4 DNA ligase and restriction buffer. Thereafter, the mixture was incubated at 16 °C for 2.5–3 h and kept at 4 °C overnight. Plasmid was transformed into *Escherichia coli* DH5α competent cells. LB broth was added to every tube and incubated at 37 °C in a shaker incubator for at least 1 h. Meanwhile, LB agar plates were warmed up at 37 °C. After centrifugation (2500 rpm for 3 min) and removal of the supernatant, the remaining mixture was spread on LB agar plates using a spreader, followed by incubation at 37 °C overnight. Recombinant clones from this transformation were then selected to be sequenced, transferred in LB broth with ampicillin (50 µg/mL) (Wako, Saitama, Japan), and incubated at 37 °C overnight in a shaker incubator. The plasmid was extracted from this culture using the Nucleospin^®^ Plasmid QuickPure (Macherey-Nagel-German) Kit.

### 4.5. Sequence Analysis

After cloning, sequence analysis was performed with the help of the Big Dye Terminator Cycle Sequencing Kit (Applied Biosystems, Waltham, MA, USA) using an ABI PRISM 3100 Genetic Analyzer (Applied Biosystems, Waltham, MA, USA). Sequences obtained in the present study were deposited to the GenBank database of the National Center for Biotechnology Information using BankIt. The GenBank accession numbers were assigned as follows: MT337501, MT337502 for *B. ovis*; MT337516, MT337517 for *T. ovis*; MT344080, MT344081, and MT344082 for *A. ovis*; and MT337504, MT337505, MT337506, MT337507, and MT337508 for *A. phagocytophilum*.

### 4.6. Phylogenetic Analysis

Nucleotide sequence similarities and identities were determined by using BLASTn analysis. The Neighbor-joining and Maximum likelihood methods of the MEGA version 7 software were employed to construct phylogenetic trees. Bootstrap analysis with 1000 replication was used to estimate the confidence of the nodes and branches of the trees.

### 4.7. Ethical Statement

The owners of the sheep were informed about the study, and their approval was obtained for sampling. All experimental procedures were carried out according to the ethical guidelines for the use of animal samples permitted by Obihiro University of Agriculture and Veterinary Medicine (Approval ID: 18–41).

### 4.8. Statistical Analysis

A cross-tabulation evaluation was made using categorical data, numbers, and percentages. In cases where the expected cells fell below 20%, the data were determined using the Monte Carlo Simulation Method for inclusion in the analysis. The significance level was indicated to be α = 0.05. The SPSS 25 (IBM Corp. Released 2017. IBM SPSS Statistics for Windows, Version 25.0. Armonk, NY: IBM Corp.) statistical package program was adopted to analyze the data. *p*-Values were computed to determine the level of statistical significance between provinces according to the obtained data.

## Figures and Tables

**Table 1:** Global prevalence of tick-borne pathogens (TBPs) identified in the study.

	*B. ovis*	*T. ovis*	*T. lestoquardi*	*A. ovis*	*A. phagocytophilum*
	n/N/%	n/N/%	n/N/%	n/N/%	n/N/%
Afyon	0/20/0	13/20/65	0/20/0	1/20/5	12/20/60
Ankara	1/20/5	13/20/65	0/20/0	4/20/20	14/20/70
Aydın	0/20/0	9/20/45	0/20/0	2/20/10	10/20/50
Bartın	1/20/5	7/20/35	0/20/0	5/20/25	12/20/60
Batman	1/19/5.26	15/19/78.95	0/19/0	9/19/47.37	8/19/42.11
Çorum	1/20/5	9/20/45	0/20/0	2/20/10	15/20/75
Elazığ	0/20/0	8/20/40	0/20/0	0/20/0	14/20/70
Iğdır	0/20/0	1/20/5	0/20/0	2/20/10	7/20/35
Isparta	1/20/5	13/20/65	0/20/0	2/20/10	12/20/60
Karabük	0/20/0	5/20/25	0/20/0	3/20/15	15/20/75
Kars	0/20/0	1/20/5	0/20/0	1/20/5	10/20/50
Kırşehir	2/20/10	13/20/65	0/20/0	5/20/25	11/20/55
Mardin	0/20/0	12/20/60	0/20/0	7/20/35	14/20/70
Niğde	1/20/5	5/20/25	0/20/0	4/20/20	17/20/85
Samsun	0/20/0	0/20/0	0/20/0	1/20/5	0/20/0
Total	8/299/2.68	124/299/41.47	0/299/0	48/299/16.05	171/299/57.19

n: number of positive samples, N: the total number of tested samples, %: prevalence of infection.

**Table 2:** Distribution of mixed infections in sheep blood samples.

	Single Infections (n/%)					Dual Infections (n/%)					Triple Infections (n/%)			Quadruple Infections (n/%)	Total (n/%)
	*Bo*	*To*	*Tl*	*Ao*	*Ap*	*Bo + To*	*Bo + Ap*	*To + Ao*	*To + Ap*	*Ao + Ap*	*Bo + To + Ao*	*Bo + To + Ap*	*To + Ao + Ap*	*Bo + To + Ao + Ap*	
Afyon	-	4/20	-	-	4/20	-	-	1/5	8/40	-	-	-	-	-	17/85
Ankara	-	3/15	-	-	4/20	-	1/5	1/5	6/30	-	-	-	3/15	-	18/90
Aydın	-	2/10	-	-	4/20	-	-	1/5	5/25	-	-	-	1/5	-	13/65
Bartın	-	1/5	-	1/5	5/25	-	-	-	3/15	1/5	-	-	2/10	1/5	14/70
Batman	-	5/26.3	-	3/15.8	1/5.3	-	-	3/15.8	3/15.8	-	-	1/5.3	3/15.8	-	19/100
Çorum	-	-	-	-	7/35	-	-	1/5	6/30	-	-	1/5	1/5	-	16/80
Elazığ	-	2/10	-	-	8/40	-	-	-	6/30	-	-	-	-	-	16/80
Iğdır	-	-	-	2/10	6/30	-	-	-	1/5	-	-	-	-	-	9/45
Isparta	-	3/15	-	-	4/20	-	-	1/5	8/40	-	1/5	-	-	-	17/85
Karabük	-	1/5	-	-	10/50	-	-	-	2/10	1/5	-	-	2/10	-	16/80
Kars	-	-	-	1/5	9/45	-	-	-	1/5	-	-	-	-	-	11/55
Kırşehir	-	2/10	-	1/5	2/10	-	-	1/5	7/35	-	1/5	-	1/5	1/5	16/80
Mardin	-	2/10	-	1/5	4/20	-	-	-	4/20	-	-	-	6/30	-	17/85
Niğde	-	-	-	-	14/70	1/5	-	-	-	-	-	-	4/20	-	19/95
Samsun	-	-	-	1/5	-	-	-	-	-	-	-	-	-	-	1/5
Total	-/0	25/8.36	-/0	10/3.34	82/27.42	1/0.33	1/0.33	9/3.01	60/20.07	2/0.67	2/0.67	2/0.67	23/7.69	2/0.67	219/73.24

n: number of positive samples, %: prevalence of infection, *Bo*: *Babesia ovis*, *To*: *Theileria ovis*, *Tl*: *Theileria lestoquardi*, *Ao*: *Anaplasma ovis*, *Ap*: *Anaplasma phagocytophilum*.

**Table 3:** Provincial comparison of molecular prevalences of tick-borne pathogens.

		Niğde	Iğdır	Aydın	Mardin	Bartın	Kars	Samsun	Afyon	Elazığ	Karabük	Çorum	Ankara	Isparta	Kırşehir	Batman	Total
***A. ovis***	N	16_a,b,c,d_	18_b,d,e_	18_b,d,e_	13_c,d_	15_a,b,c,d_	19_b,e_	19_b,e_	19_b,e_	20_e_	17_b,d,e_	18_b,d,e_	16_a,b,c,d_	18_b,d,e_	15_a,b,c,d_	10_a,c_	251
	%	80	90	90	65	75	95	95	95	100	85	90	80	90	75	52.6	83.9
	P	4_a,b,c,d_	2_b,d,e_	2_b,d,e_	7_c,d_	5_a,b,c,d_	1_b,e_	1_b,e_	1_b,e_	0_e_	3_b,d,e_	2_b,d,e_	4_a,b,c,d_	2_b,d,e_	5_a,b,c,d_	9_a,c_	48
	%	20	10	10	35	25	5	5	5	0	15	10	20	10	25	47.4	16.1
***A. phagocytophilum***	N	3_a_	13_b_	10_b,c,d_	6_a,c,d_	8_a,b,c,d_	10_b,c,d_	20_e_	8_a,b,c,d_	6_a,c,d_	5_a,d_	5_a,d_	6_a,c,d_	8_a,b,c,d_	9_b,c,d_	11_b,c_	128
	%	15	65	50	30	40	50	100	40	30	25	25	30	40	45	57.9	42.8
	P	17_a_	7_b_	10_b,c,d_	14_a,c,d_	12_a,b,c,d_	10_b,c,d_	0_e_	12_a,b,c,d_	14_a,c,d_	15_a,d_	15_a,d_	14_a,c,d_	12_a,b,c,d_	11_b,c,d_	8_b,c_	171
	%	85	35	50	70	60	50	0	60	70	75	75	70	60	55	42.1	57.2
***B. ovis***	N	19_a_	20_a_	20_a_	20_a_	19_a_	20_a_	20_a_	20_a_	20_a_	20_a_	19_a_	19_a_	19_a_	18_a_	18_a_	291
	%	95	100	100	100	95	100	100	100	100	100	95	95	95	90	94.7	97.3
	P	1_a_	0_a_	0_a_	0_a_	1_a_	0_a_	0_a_	0_a_	0_a_	0_a_	1_a_	1_a_	1_a_	2_a_	1_a_	8
	%	5	0	0	0	5	0	0	0	0	0	5	5	5	10	5.3	2.7
***T. ovis***	N	15_a,b,c,d,e,f,g_	19_f,g,h_	11_d,e,i,j,k,l_	8_k,l,m_	13_c,e,i,j,k,l_	19_b,g,h_	20_h_	7_j,l,m_	12_a,c,d,e,i,j,k,l_	15_a,b,c,d,e,f,g_	11_a,c,d,e,i,j,k,l_	7_i,j,k,l,m_	7_i,j,k,l,m_	7_i,j,k,l,m_	4_m_	175
	%	75	95	55	40	65	95	100	35	60	75	55	35	35	35	21.1	58.5
	P	5_a,b,c,d,e,f,g_	1_f,g,h_	9_d,e,i,j,k,l_	12_k,l,m_	7_c,e,i,j,k,l_	1_b,g,h_	0_h_	13_j,l,m_	8_a,c,d,e,i,j,k,l_	5_a,b,c,d,e,f,g_	9_a,c,d,e,i,j,k,l_	13_i,j,k,l,m_	13_i,j,k,l,m_	13_i,j,k,l,m_	15_m_	124
	%	25	5	45	60	35	5	0	65	40	25	45	65	65	65	78.9	41.5
***T. lestoquardi***	N	20_a_	20_a_	20_a_	20_a_	20_a_	20_a_	20_a_	20_a_	20_a_	20_a_	20_a_	20_a_	20_a_	20_a_	19_a_	299
	%	100	100	100	100	100	100	100	100	100	100	100	100	100	100	100	100
	P	0_a_	0_a_	0_a_	0_a_	0_a_	0_a_	0_a_	0_a_	0_a_	0_a_	0_a_	0_a_	0_a_	0_a_	0_a_	0_a_
	%	0	0	0	0	5	0	0	0	0	0	0	0	0	0	0	0

N: number of negate samples, P: number of positive samples, %: prevalence value. Each subscript letter denotes a subset of province categories whose column proportions do not differ significantly from each other.

**Table 4:** Primer pairs used in the standard and nPCR assays for detection of each pathogen.

Species	Target Gene	Primer Name	Primer Sequence (5′…′3)	Expected Size (bp)	Annealing Temperature (°C)	References
*B. ovis*	SSU rRNA	Bov F	TGGGCAGGACCTTGGTTCTTCT	549 bp	62	Aktas et al. [32]
		Bov R	CCGCGTAGCGCCGGCTAAATA			
*T. ovis*	SSU rRNA	TSsr 170-F	TCGAGACCTTCGGGT	520 bp	60	Aktas et al. [52]
		TSsr 670-R	TCCGGACATTGTAAAACAAA			
*T. lestoquardi*	18S rRNA	*T. lestoquardi* F	GTGCCGCAAGTGAGTCA	730 bp	52	Kirvar et al. [53]
		*T. lestoquardi* R	GGACTGATGAGAAGACGATGAG			
*A. ovis*	*MSP-4*	MSP-4-F	TGAAGGGAGCGGGGTCATGGG	347 bp	62	Torina et al. [54]
		MSP-4R	GAGTAATTGCAGCCAGGCACTCT			
*A. phagocytophilum*	16S rRNA	EE1	TCCTGGCTCAGAACGAACGCTGGCGGC	1433 bp	50	Barlough et al. [55]
		EE2	AGTCACTGACCCAACCTTAAATGGCTG			
		SSAp-F	GCT GAA TGT GGG GAT AAT TTA T	641 bp	55	Kawahara et al. [56]
		SSAp-R	ATG GCT GCT TCC TTT CGG TTA			

## Data Availability

The authors confirm that the data supporting the findings of this study are available within the article.

## References

[1] Uilenberg G. (1995). International collaborative research: Significance of tick-borne hemoparasitic diseases to world animal health. Vet. Parasitol..

[2] Sevinc F., Xuan X. (2015). Major tick-borne parasitic diseases of animals: A frame of references in Turkey. Eurasian J. Vet. Sci..

[3] Stuen S., Bergstrom K., Palmer E. (2002). Reduced weight gain due to subclinical *Anaplasma phagocytophilum* (formerly *Ehrlichia phagocytophila*) infections. Exp. Appl. Acarol..

[4] Garcia-Perez A.L., Barandika J., Oporto B., Povedano I., Juste R.A. (2003). *Anaplasma phagocytophila* as an abortifacient agent in sheep farms from northern Spain. Ann. N. Y. Acad. Sci..

[5] Stuen S. (2016). Haemoparasites in small ruminants in European countries: Challenges and clinical relevance. Small Rumin. Res..

[6] Aouadi A., Leulmi H., Boucheikhchoukh M., Benakhla A., Raoult D., Parola P. (2017). Molecular evidence of tick-borne hemoprotozoan-parasites (*Theileria ovis* and *Babesia ovis*) and bacteria in ticks and blood from small ruminants in Northern Algeria. Comp. Immunol. Microbiol. Infect. Dis..

[7] Oluwatayo I.B., Oluwatayo T.B. (2012). Small ruminants as a source of financial security: A case study of woman in rural Southwest Nigeria. Inst. Money Technol. Financ. Incl..

[8] Turkısh Statistical Institute. http://www.turkstat.gov.tr/.

[9] Sevinc F., Sevinc M., Ekici O.D., Yildiz R., Isik N., Aydogdu U. (2013). *Babesia ovis* infections: Detailed clinical and laboratory observations in the pre- and post-treatment periods of 97 field cases. Vet. Parasitol..

[10] Alessandra T., Santo C. (2012). Tick-borne diseases in sheep and goats: Clinical and diagnostic aspects. Small Rumin. Res..

[11] Sparagano O.A.E., Spitalska E., Namavari M., Torina A., Cannella V., Caracappa S. (2006). Phylogenetics of *Theileria* species in small ruminants. Ann. N. Y. Acad. Sci..

[12] Razmi G., Pourhosseini M., Yaghfouri S., Rashidi A., Seidabadi M. (2013). Molecular detection of *Theileria* spp. and *Babesia* spp. in sheep and ixodid ticks from the northeast of Iran. J. Parasitol..

[13] Renneker S., Abdo J., Bakheit M.A., Kullmann B., Beyer D., Ahmed J., Seitzer U. (2013). Co-infection of sheep with *Anaplasma*, *Theileria* and *Babesia* species in the Kurdistan region, Iraq. Transbound. Emerg. Dis..

[14] Bilgic H.B., Bakirci S., Kose O., Unlu A.H., Hacilarlioglu S., Eren H., Weir W., Karagenc T. (2017). Prevalence of tick-borne haemoparasites in small ruminants in Turkey and diagnostic sensitivity of single-PCR and RLB. Parasit. Vectors..

[15] De la Fuente J., Atkinson M.W., Naranjo V., Fernandez de Mera I.G., Mangold A.J., Keating K.A., Kocan K.M. (2007). Sequnce analysis of the *msp4* gene of *Anaplasma ovis* strains. Vet. Microbiol..

[16] Chochlakis D., Koliou M., Ioannou I., Tselentis Y., Psaroulaki A. (2009). Kawasaki disease and *Anaplasma* sp. infection of an infant in Cyprus. Int. J. Infect. Dis..

[17] Chochlakis D., Ioannou I., Tselentis Y., Psaroulaki A. (2010). Human anaplasmosis and *Anaplasma ovis* variant. Emerg. Infect. Dis..

[18] Stuen S. (2013). Tick-borne infections in small ruminants in northern Europe. Small Rumin. Res..

[19] Stuen S., Granquist E.G., Silaghi C. (2013). *Anaplasma phagocytophilum*- a widespread multi-host pathogen with highly adaptive strategies. Front. Cell. Infect. Microbiol..

[20] Inci A., Ica A., Yildirim A., Duzlu O. (2010). Identification of *Babesia* and *Theileria* species in small ruminants in Central Anatolia (Turkey) via reverse line blotting. Turk. J. Vet. Anim. Sci..

[21] Ozubek S., Aktas M. (2017). Molecular and parasitological survey of ovine piroplasmosis, including the first report of *Theileria annulata* (Apicomplexa: Theileridae) in sheep and goats from Turkey. J. Med. Entomol..

[22] Zhou M., Cao S., Sevinc F., Sevinc M., Ceylan O., Ekici S., Jirapattharasate C., Moumouni P.F.A., Liu M., Wang G. (2017). Molecular detection and genetic characterization of *Babesia*, *Theileria* and *Anaplasma* amongst apparently healthy sheep and goats in the central region of Turkey. Ticks Tick Borne Dis..

[23] Sevinc F., Zhou M., Cao S., Ceylan O., Aydin M.F., Sevinc M., Xuan X. (2018). Haemoparasitic agents associated with ovine babesiosis: A possible negative interaction between *Babesia ovis* and *Theileria ovis*. Vet. Parasitol..

[24] Ringo A.E., Moumouni P.F.A., Taioe M., Jirapattharasate C., Liu M., Wang G., Gao Y., Guo H., Lee S., Zheng W. (2018). Molecular analysis of tick-borne protozoan and rickettsial pathogens in small ruminants from two South African provinces. Parasitol. Int..

[25] De la Fuente J., Estrada-Pena A., Venzal J.M., Kocan K.M., Sonenshine D.E. (2008). Overview: Ticks as vectors of pathogens that cause disease in humans and animals. Front. Biosci..

[26] Yin H., Schnittger L., Luo J., Seitzer U., Ahmed J.S. (2007). Ovine theileriosis in China: A new look at an old story. Parasitol. Res..

[27] Bai Q., Liu G., Liu D., Ren J., Li X. (2002). Isolation and preliminary characterization of a large *Babesia* sp. from sheep and goats in the eastern part of Gansu Province, China. Parasitol. Res..

[28] Uilenberg G. (2006). Babesia—A historical overview. Vet. Parasitol..

[29] Liu A.H., Yin H., Guan G.Q., Schnittger L., Liu Z.J., Ma M.L., Dang Z.S., Liu J.L., Ren Q.Y., Bai Q. (2007). At least two genetically distinct large *Babesia* species infective to sheep and goats in China. Vet. Parasitol..

[30] Ceylan O., Sevinc F. (2020). Endemic instability of ovine babesiosis in Turkey: A country-wide sero-epidemiological study. Vet. Parasitol..

[31] Aydin M.F., Dumanli N. (2019). Tick-borne pathogens in small ruminants in Turkey: A systematic review. Turk. Vet. J..

[32] Aktas M., Altay K., Dumanli N. (2005). Development of a polymerase chain reaction method for diagnosis of *Babesia ovis* infection in sheep and goat. Vet. Parasitol..

[33] Altay K., Dumanli N., Aktas M. (2012). A study on ovine tick-borne hemoprotozoan parasites (*Theileria* and *Babesia*) in the East Black Sea Region of Turkey. Parasitol. Res..

[34] Karatepe B., Ozubek S., Karatepe M., Aktas M. (2019). Detection of *Theileria* and *Babesia* species in sheep and goats by microscopy and molecular methods in Nigde province, Turkey. Revue Med. Vet..

[35] Ahmed J., Yin H., Bakheit M., Liu Z., Mehlhorn H., Seitzer U., Mehlhorn H. (2011). Small ruminant theileriosis. Progress in Parasitology.

[36] Friedhoff K.T. (1997). Tick-borne disease of sheep and goats caused by *Babesia*, *Theileria* or *Anaplasma* spp. Parassitologia.

[37] Altay K., Dumanli N., Holman P.J., Aktas M. (2005). Detection of *Theileria ovis* in naturally infected sheep by nested PCR. Vet. Parasitol..

[38] Ringo A.E., Aboge G., Moumouni P.F.A., Lee S.H., Jirapattharasate C., Liu M., Gao Y., Guo H., Zheng W., Efstratiou A. (2019). Molecular detection and genetic characterisation of pathogenic *Theileria*, *Anaplasma* and *Ehrlichia* species among apparently healthy sheep in central and western Kenya. Onderstepoort J. Vet. Res..

[39] Renneker S., Abdo J., Salih D.E.A., Karagenc T., Bilgic H., Torina A., Oliva A.G., Campos J., Kullmann B., Ahmed J. (2013). Can *Anaplasma ovis* in small ruminants be neglected any longer?. Transbound. Emerg. Dis..

[40] Lbacha H.A., Alali S., Zouagui Z., El Mamoun L., Rhalem A., Petit E., Haddad N., Gandoin C., Boulouis H.J., Maillard R. (2017). High prevalence of *Anaplasma* spp. in small ruminants in Morocco. Transbound. Emerg. Dis..

[41] Altay K., Dumanli N., Aktas M., Ozubek S. (2014). Survey of *Anaplasma* infections in small ruminants from east part of Turkey. Kafkas Univ. Vet. Fak. Derg..

[42] Aktas M., Ozubek S. (2018). *Anaplasma ovis* genetic diversity detected by major surface protein 1a and its prevalence in small ruminants. Vet. Microbiol..

[43] Benedicto B., Ceylan O., Moumouni P.F.A., Lee S., Li J., Galon E.M., Liu M., Li Y., Ji S., Tumwebaze M.A. (2020). Molecular detection and assessment of risk factors for tick-borne diseases in sheep and goats from Turkey. Acta Parasitol..

[44] Oter K., Cetinkaya H., Vurusaner C., Toparlak M., Ergunay K. (2016). Molecular detection and typing of *Anaplasma* species in small ruminants in Thrace Region of Turkey. Kafkas Univ. Vet. Fak. Derg..

[45] Unver A., Sahin M., Erdogan H.M., Celebi O. (2005). Investigation of antibodies against *Anaplasma phagocytophilum* in sheep by western blot analyses. Kafkas Univ. Vet. Fak. Derg..

[46] Gokce H.I., Genc O., Akca A., Vatansever Z., Unver A., Erdogan H.M. (2008). Molecular and serological evidence of *Anaplasma phagocytophilum* infection of farm animals in the Black Sea Region of Turkey. Acta Vet. Hung..

[47] Aktas M., Vatansever Z., Altay K., Aydin M.F., Dumanli N. (2010). Molecular evidence for *Anaplasma phagocytophilum* in *Ixodes ricinus* from Turkey. Trans. R. Soc. Trop. Med. Hyg..

[48] Aktas M., Altay K., Ozubek S., Dumanli N. (2012). A survey of ixodid ticks feding on cattle and prevalence of tick-borne pathogens in the Black Sea region of Turkey. Vet. Parasitol..

[49] Aktas M. (2014). A survey of ixodid tick species and molecular identification of tick-borne pathogens. Vet. Parasitol..

[50] Giangaspero A., Marangi M., Papini R., Paoletti B., Wijnveld M., Jongejan F. (2015). *Theileria* sp. OT3 and other tick-borne pathogens in sheep and ticks in Italy: Molecular characterization and phylogeny. Ticks Tick Borne Dis..

[51] Rjeibi M.R., Gharbi M., Mhadhbi M., Mabrouk W., Ayari B., Nasfi I., Jedidi M., Sassi L., Rekik M., Darghouth M.A. (2014). Prevalence of piroplasms in small ruminants in North-West Tunisia and the first genetic characterisation of *Babesia ovis* in Africa. Parasite.

[52] Aktas M., Altay K., Dumanli N. (2006). PCR-based detection of *Theileria ovis* in *Rhipicephalus bursa* adult ticks. Vet. Parasitol..

[53] Kirvar E., Wilkie G., Katzer F., Brown C.G.D. (1998). *Theileria lestoquardi*–maturation and quantification in *Hyalomma anatolicum anatolicum* ticks. Parasitology.

[54] Torina A., Agnone A., Blanda V., Alongi A., D’Agostino R., Caracappa S., de la Fuente J. (2012). Development and validation of two PCR tests for the detection of and differentiation between *Anaplasma ovis* and *Anaplasma marginale*. Ticks Tick Borne Dis..

[55] Barlough J.E., Madigan J.E., DeRock E., Dumler J.S., Bakken J.S. (1995). Protection against *Ehrlichia equi* is conferred by prior infection with the human granulocytic ehrlichia (HGE agent). J. Clin. Microbiol..

[56] Kawahara M., Rikihisa Y., Lin Q., Isogai E., Tahara K., Itagaki A., Hiramitsu Y., Tajima T. (2006). Novel genetic variants of *Anaplasma phagocytophilum*, *Anaplasma bovis*, *Anaplasma centrale*, and a novel *Ehrlichia* sp. in wild deer and ticks on two major islands in Japan. Appl. Environ. Microbiol..

